# The Buccal Protozoa

**Published:** 1914-10-15

**Authors:** Edward C. Kirk


### THE BUCCAL PROTOZOA.

BY EDWARD C. KIRK, D.D.S.

Two important communications upon the protozoa of the buccal cavity have recently been made, both of which place the vexed question of the etiology of so-called pyorrhea alveolaris in a new and interesting light;—the first by Prof. Allen J. Smith and Dr. M. T. Barrett, read before the recent meeting of the Pennsylvania State Dental Society in Philadelphia, Pa. (June 30th to July 2d), and published in full in our August issue, at page 948, and the second by Prof. Angelo Chiavaro of Rome, Italy, read before the Paris meeting of the American Dental Society of Europe on July 30th, an abstract of which paper is given at page 1089 of the present issue of the *Cosmos.*

The paper by Professor Chiavaro is essentially a biological study of the two principal varieties of protozoa thus far identified as inhabitants of the oral cavity, viz, *Endamoeba buccalis* and *Flagellata hominis.* It is, however, significant that of the sixty-eight specimens examined from the same number of individual cases, twenty-two were pyorrhetic, and in all these latter without exception endamoeba were found abundantly, the protozoon being also found in fourteen other cases in small number, leaving a total of thirty-two out of sixty-eight cases examined in which the organism was absent.

It is also worthy of note that the endamoeba was not found in carious cavities where the carious process was in active progress, due doubtless to the observed fact that the endamoeba dies in an acid medium. The practically constant presence of these protozoal organisms in the pus exudate in pyorrheal cases observed also by Prof. Allen J. Smith and Dr. M. T. Barrett led the former to suggest a test of emetin hydrochlorid as a topical application in pyorrheal treatment, upon the assumption that the well-established toxicity of ipecacuanha to the amoeba coli, as amply demonstrated by its successful use in the treatment of amoebic dysentery, might be of value in destroying the vitality of the morbific agent in pyorrhea, assuming further that the protozoa under consideration were the exciters of the inflammatory process in pyorrhea alveolaris.

The practical test of the treatment by emetin hydrochlorid as reported by Drs. Smith and Barrett has given results that are encouraging, not to say astonishing. And since the announcement of these results further tests made by several careful and conservative observers in Germany, France, and England have thus far fully corroborated the findings of Drs. Smith and Barrett.

The method of procedure adopted in the clinical tests made in Europe is to cleanse all pus pockets by pressure and irrigation with physiological salt solution or distilled water, so as to eliminate as fully as possible all pus exudate, and then to instil, by means of a blunt-pointed hypodermic syringe, or other suitable applicator, a few drops of a one per cent. solution of emetin hydrochlorid into the pockets, anointing their margins with petrolatum or vaseline, with a view to retaining the solution in contact with the pocket walls as long as possible. The treatment is to be repeated every other day until all pus flow and inflammation subsides, when thorough instrumentation for the removal of deposits upon the root surfaces may be instituted.

If the one per cent. solution of emetin is found to be locally irritating it may be largely diluted, as the drug is known to be fatally toxic to amoeba in a dilution of 1:10,000 and after some minutes' exposure they will die in a solution as dilute as 1:100,000. The drug is not toxic to man and does not produce nausea when injected hypodermically in doses of one-third grain (0.02 gm.), and as much as one-half grain has been injected without producing unpleasant effects. When taken into the stomach it provokes active emesis.

In the present stage of the investigation it is hazardous to draw general conclusions as to the relations, if any, which the endamoeba buccalis or the flagellata hominis may bear to the etiology of pyrrhea alveolaris, or if, indeed, these protozoa are possessed of pathogenic properties at all. That important question will require much further careful study before it can be determined positively or negatively. The destructive effect which emetin exerts upon the buccal protozoa is clearly demonstrated, yet it is known that the drug is destructive of the vitality of certain bacterial organisms, and is therefore not specific in its toxic action upon protozoa. But the study of the subject thus far has brought out the important practical fact that emetin appears to have in pyorrhea treatment a therapeutic action of far greater efficiency than any other medicament known to dental materia medica, and the further scientific study of its action must undoubtedly reveal in time some of the fundamental causes of this puzzling group of disorders so destructive to the integrity of the masticating machanism and to the general bodily health.—*Dental Cosmos*, Sept. 1914.

